# Task instructions can accelerate the early preference for social features in naturalistic scenes

**DOI:** 10.1098/rsos.180596

**Published:** 2019-03-06

**Authors:** Albert End, Matthias Gamer

**Affiliations:** 1 Department of Systems Neuroscience, University Medical Centre Hamburg-Eppendorf, Hamburg, Germany; 2 Department of Psychology, Julius Maximilians University of Würzburg, Würzburg, Germany

**Keywords:** social attention, top-down influence, task effects, naturalistic scenes, physical saliency, eye movements

## Abstract

Previous research demonstrated that humans rapidly and reflexively prioritize social features (especially heads and faces) irrespective of their physical saliency when freely viewing naturalistic scenes. In the current study, we investigated whether this preference for social elements already occurs maximally fast during free exploration or whether it is possible to additionally accelerate it by means of top-down instructions. To examine this question, we presented participants with colour photographs of naturalistic scenes containing social features (e.g. heads and bodies) while recording their eye movements. For half of the stimuli, observers were instructed to freely view the images; for the other half of the stimuli, their task was to spot depicted people as fast as possible. We replicated that social elements (especially heads) were rapidly preferred over physically salient image parts. Moreover, we found the orienting towards social elements to be additionally enhanced and accelerated when participants were instructed to detect people quickly. Importantly, this effect was strongest for heads and already evident at the very first fixation. Thus, the present study not only corroborates that the prioritization of social features in naturalistic scenes partially relies on reflexive processes, but also demonstrates that these mechanisms can be additionally accelerated by top-down instructions.

## 1. Introduction

In daily life, humans constantly come across other people, interact with them and reciprocally signal various kinds of cues, for instance regarding their current emotional state (e.g. by means of facial expressions) or their momentary focus of attention (e.g. by means of head or gaze direction). Hence, there have been innumerable face perception studies which used rather simplistic visual stimuli (i.e. isolated faces) to investigate the processing of such social signals (e.g. [1–4]). However, natural human vision typically comprises diverse kinds of elements across the whole visual field which directly compete for the limited attention and processing resources. Thus, before being able to process specific features of faces of others, it is essential to direct attention to fellow humans in the first place [5–7]. Accordingly, there is a growing number of studies relying on more naturalistic visual material. In such stimuli, social features, such as faces, heads or bodies, are depicted as only one of several aspects within surrounding environments (e.g. in the street). Importantly, this approach provided strong evidence that observers preferentially allocate their attention to other people (especially their heads and eyes) as compared to non-social visual information (e.g. [5,8–11]). This research further indicated that this attentional preference for other people was evident even if the competing non-social information was highly conspicuous regarding low-level features (e.g. [10]). Moreover, several studies yielded support that this bias is present and probably even most pronounced for the very first fixations after stimulus onset indicating that it does not just emerge after several seconds of visual exploration (e.g. [10,12–15]). This notion is further corroborated by a recent study [16] which demonstrated a preference of first eye movements for those regions of naturalistic scenes that contained human beings. Crucially, the social preference occurred in this study even though the very brief stimulus presentation time of 200 ms precluded further scene exploration and was too short to allow for voluntary gaze shifts ([16]; see also [17]). This research suggests that the attentional prioritization of social features in naturalistic scenes does not only rely on volitionally controlled mechanisms, but especially reflects the influence of reflexive and automatic processes. However, the very rapid phenomenon of social orienting has still not been characterized sufficiently. For example, it is an interesting, yet unresolved research question whether this rapid bias already occurs maximally fast when observers freely explore naturalistic scenes or whether it is possible to additionally accelerate it by means of top-down instructions.

In general, the influence of top-down demands on processing social information in complex scenes has been investigated in a number of prior studies (e.g. [18–24]). This research indicates that different task instructions have the potential to either enhance (e.g. the task to determine where depicted people are directing their attention to) or diminish (e.g. the task to spot the depicted location) the degree of orienting towards social features. However, the majority of previous studies made no systematic differentiation between early and late viewing stages. Thus, the potential influence of top-down demands on the preferential scanning of people in visual scenes has so far not been explored sufficiently for the very first eye movements. Importantly, it was shown that the preference for social features in naturalistic scenes already occurs extremely fast under free viewing conditions and is most pronounced for the very first fixations (e.g. [10,12–15]). Hence, it is indispensable to focus on early viewing stages to investigate whether the rapid orienting towards social information can be further speeded up by task instructions. In detail, this research question can be answered only by exploring the extent and latency of very first fixations directed towards social elements. Interestingly, there is some support that task instructions may have the potential to slightly decrease the number of early fixations on social elements in naturalistic scenes (e.g. [25–27]). Contrary to this, the few studies which examined whether their general finding of top-down induced enhancement of orienting towards humans would also be present for early eye movements yielded partly inconsistent results (e.g. [28–30]). For example, Fletcher-Watson and colleagues [29,30] presented their participants with composite stimuli consisting of a social and a non-social scene and instructed them either to view the stimuli freely or to judge the gender of a depicted person. This research provided support that participants directed more first fixations to the social scene in general and specifically to the depicted person when judging gender as compared to free exploration. By contrast, a study by Birmingham *et al*. [28] demonstrated task-dependent modulations of the preference for humans (especially their eyes) in naturalistic scenes only across the whole viewing duration of 15 s. In detail, the preference was more pronounced when participants had to describe the depicted actors’ foci of attention as compared to generally describing the image or freely exploring it. However, there was no evidence for similar task-dependent modulations at early fixations.

Moreover, most previous studies exploring potential influences of task instructions on rapid orienting towards socially relevant information in visual scenes did not sufficiently control for differences between social and non-social image regions in low-level stimulus properties. However, traditional theories postulate attention to be driven both by top-down demands and by bottom-up processing of stimulus characteristics [31–33]. In addition, computational models within this framework postulate that attention can be predicted by so-called physical saliency (e.g. [34,35]; for reviews see [36–38]). In detail, these models assume attention to be particularly allocated to those aspects of the visual field which are conspicuous by differing from their surrounding with respect to physical features (e.g. colour, intensity or orientation). In this context, the physical saliency is defined by the amount that each location of the visual input stands out from the background in terms of predefined low-level characteristics. For example, in the traditional model of Itti and Koch [34,35], a computational algorithm calculates the physical saliency for all locations of a visual input image using biologically plausible centre-surround differences regarding the features colour, intensity and orientation. First, conspicuous locations are calculated separately for each feature. Subsequently, the calculations for all features are combined into an overall map indicating the physical saliency of each location of the visual input. Following the seminal model of Itti and Koch, a multitude of saliency algorithms has been developed (for reviews see [36–38]). These models differ in the computational approaches used to determine physical saliency as well as in the low-level features they are based on.

Importantly, previous studies yielded support for the power of saliency-based approaches in predicting the allocation of attention during scene viewing (e.g. [39]) and indicated that saliency might be especially important at early viewing stages (e.g. [40]; but see [41]). This demonstrates that humans can exhibit a tendency to orient towards physically conspicuous aspects of visual input. Therefore, to ensure that participants really allocate their attention to certain visual locations because of the high-level information that is displayed in these regions (e.g. heads or faces of conspecifics), it is necessary to avoid confounds between the high-level information of interest and low-level physical saliency. It has to be mentioned that there is evidence that the influence of low-level saliency on eye movements is reduced under certain tasks (e.g. [42–44]) and for socially relevant stimuli (e.g. [10–12,45]). Nevertheless, an essential aspect of research examining the attentional preference for humans in visual scenes, which has been increasingly taken into consideration in recent studies (e.g. [10,27]), is to control for the physical saliency of social and non-social image elements. For example, a study revealing top-down enhancements of rapid orienting towards persons could not unambiguously interpret this finding without sufficiently ruling out that depicted persons are physically highly salient at the same time. Without controlling for this confound, the study could not disentangle whether participants were indeed able to increase early social attention or just the processing of highly salient elements.

**Figure 1.**
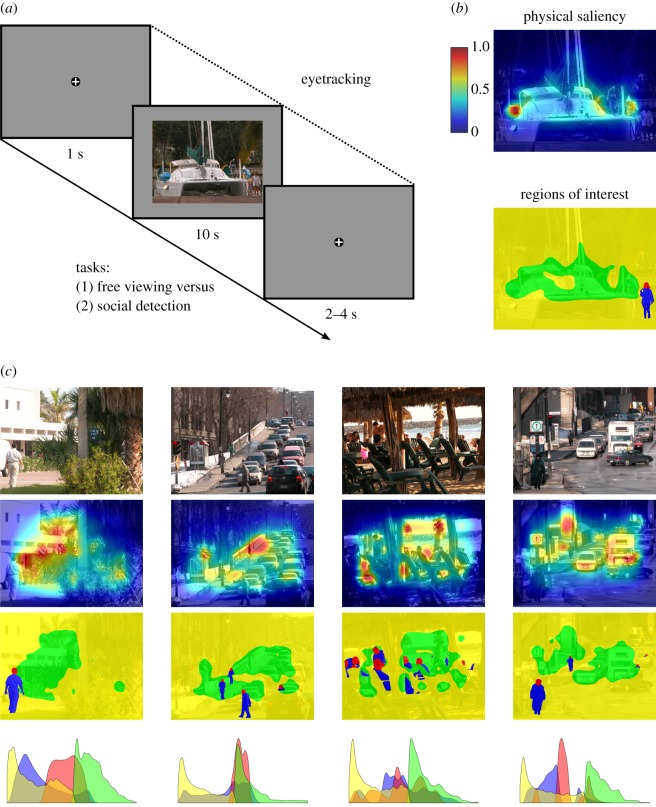
Methods. (*a*) Illustration of the general trial procedure. Each scene was either viewed freely or with the task to spot people as fast as possible while eye movements were recorded. Note: Size of fixation cross is not to scale. Photograph of visual scene reproduced by the kind permission of F. A. A. Kingdom [46]. (*b*) Heat map for physical saliency (cold colours = low saliency, warm colours = high saliency) and regions of interest (ROIs: red = head, blue = body, yellow = area of lower saliency, green = area of higher saliency) overlaid on the corresponding example scene from figure 1*a*. Heat map for physical saliency and ROIs as published (with partly different colouring) in End and Gamer (2017) [10]. The colour bar key for physical saliency is valid for all heat maps of physical saliency in this figure. (*c*) Further examples of original scene images with the corresponding heat maps for physical saliency as well as the corresponding regions of interest (ROIs) and density plots depicting the distribution of physical saliency values as a function of ROI. Note: Photographs of visual scenes reproduced by kind permission of F. A. A. Kingdom [46].

In addition, it has to be noted that in a few previous studies task instructions were applied which indirectly^1^ required to attend to depicted people (e.g. gender discrimination or determination of the depicted persons' attentional foci). By contrast, to the best of our knowledge, there was no study using the direct instruction to spot people in naturalistic scenes as fast as possible to comprehensively investigate the following research question: Could the early preference of orienting towards humans (versus highly salient non-social elements) be accelerated as compared to free viewing? To close these gaps, in the current study, we presented participants with colour photographs of complex naturalistic social scenes (i.e. including human beings), while their eye movements were recorded (figure 1). In the first half of the experiment, participants were instructed to freely view each stimulus. In the second half of the experiment, they had the task to spot people in each visual scene as quickly as possible. Scene assignment to blocks was randomized for each participant while ensuring for an equal distribution of emotionally valent scenes in each block (for further details, see the Methods section). Importantly, we took care during stimulus selection that social elements were not confounded by physical saliency. Furthermore, we explicitly compared the low-level saliency between social and non-social image regions. This allowed disentangling the influence of social attention from physical saliency. Moreover, it facilitated investigating whether top-down instructions have the potential to enhance the degree that very first eye movements were directed to humans as compared to highly conspicuous non-social regions. To answer this research question, we conducted detailed analyses of the first five fixated scene locations and examined the latency after stimulus onset until different scene regions were fixated for the first time.

## 2. Methods

### 2.1. Participants

An *a priori* power analysis performed with the software G*Power (v. 3.1.9.2 [47]) revealed that a sample size of 31 or larger was required for detecting medium effects (*d_z_* = 0.50)^2^ in one-tailed paired comparisons with a power of at least 0.85. To account for potential dropouts, 34 volunteers were examined. One participant was excluded from all data analyses due to reporting a history of psychiatric illness. Therefore, the final sample consisted of 33 volunteers (22 females, mean age: 24.9 years, range: 19–35 years, s.d.: 3.5 years). All participants reported normal or corrected to normal vision, provided written informed consent and received monetary compensation. The majority were students of various disciplines (70%). None of the participants from the final sample reported to receive centrally acting medication or to have a history of neurological or psychiatric illness. The study was conducted in compliance with the Declaration of Helsinki (2008 version) and approved by the ethics committee of the German Psychological Society (DGPs).

### 2.2. Stimuli

The stimulus set comprised 80 colour photographs of complex naturalistic scenes which were already used in a previous study [10]. The photographs were collected from several databases (Emotional Picture Set, EmoPicS [49]; International Affective Picture System, IAPS [50]; McGill Calibrated Colour Image Database [46]; Nencki Affective Picture System, NAPS [51]) and the Internet (e.g. Google picture search, flickr). They portrayed diverse kinds of indoor and outdoor scenes containing mainly objects and occasionally animals. All stimuli included social features defined as (parts of) human beings (e.g. heads, bodies or body parts) and are therefore considered as social scenes (figure 1). They ranged in emotional valence from negative to positive (including neutral scenes), did not contain large pieces of conspicuous text, and were previously unknown to the participants. Using the software GIMP (v. 2.8.10, GNU Image Manipulation Program, The GIMP Team), the stimuli were cropped or rescaled to the resolution of 1200 × 900 pixels.

### 2.3. Apparatus

In a room with constant lighting conditions, a head rest guaranteed a fixed viewing distance of 52.5 cm. Each stimulus was presented on a grey background in the centre of an LCD screen (20.1″, 60 Hz) by means of the software Presentation^®^ 17.0 (Neurobehavioral Systems, Inc., Berkeley, CA, USA). The size of each stimulus amounted to 30.6 × 23.0 cm which covered a visual angle of 32.5° × 24.7°. A video-based eye tracker (EyeLink 1000, SR Research, Ottawa, ON, Canada) with a sampling rate of 1000 Hz was used to record eye movements from the right eye.

### 2.4. Procedure

Before the experiment, each participant received general information about the study, gave written informed consent, and was asked for relevant individual characteristics (e.g. defective vision, history of neurological or psychiatric illness, medication). This information was used to ensure that data analyses were performed only for participants meeting our predefined inclusion criteria (i.e. normal or corrected to normal vision, no history of neurological or psychiatric illness, not receiving centrally acting medication). This was the case for all but one volunteer (see Participants section).

The experiment consisted of two blocks, each containing 40 trials and starting with three practice trials which were excluded from data analyses. Blocks were separated by a short break. Before each block, participants were instructed about the upcoming experimental task and asked to avoid large head and body movements. In addition, before each block, the calibration and validation of the eye tracker were carried out using a nine-point grid. In each trial, participants were presented with a central fixation cross for 1 s followed by a visual scene for 10 s followed by a fixation cross for a random period between 2 and 4 s (figure 1*a*). In the first block, participants’ task was to freely view each visual scene (e.g. as if they were looking at photographs in a newspaper or magazine); in the second block, they were required to spot people in each visual scene as fast as possible. After successful detection of people in the task last mentioned, participants were allowed to freely view each visual scene. We did not require participants to indicate successful detection of people by any behavioural response (e.g. button press) in order not to interrupt visual stimulus processing. Thus, we do not know exactly at which point in time participants switched from the detection task to a free viewing mode and we therefore focused our analyses on the very first fixations after stimulus onset, when participants were most probably engaged in the spotting task. Participants were instructed to fixate the fixation cross that was presented between images in both blocks. Each participant saw all 80 stimuli but scene order within blocks as well as the assignment of scenes to blocks was randomized while ensuring variations in scene valence by balancing the selection of neutral, positive and negative scenes between blocks (for details about stimulus characteristics, see [10]).

It has to be mentioned that we did not obtain subjective ratings of emotional quality in the current study. However, we acquired ratings for each scene of the current experiment from 31 participants in one of our recent studies (see [10]). In the previous study, each scene was evaluated on 9-point scales with respect to valence, arousal and personal relevance. We used computerized versions of the Self-Assessment Manikin scales (SAM [52–54]) for valence and arousal and adopted the principle of SAM to build a customized scale for personal relevance. On this basis, the mean valence, arousal and personal relevance ratings were calculated for each scene of the current study. To ensure that emotional quality was appropriately balanced between blocks, we determined the average ratings within each block and compared them between blocks. Importantly, this revealed the blocks of free viewing and social detection to be similar regarding the rated valence (free viewing: *M* = 4.8, s.d. = 0.1, range = 4.6–4.9; social detection: *M* = 4.8, s.d. = 0.1, range = 4.6–5.0), arousal (free viewing: *M* = 4.5, s.d. = 0.1, range = 4.3–4.7; social detection: *M* = 4.5, s.d. = 0.1, range = 4.3–4.7), and personal relevance (free viewing: *M* = 3.8, s.d. = 0.1, range = 3.6–4.0; social detection: *M* = 3.9, s.d. = 0.1, range = 3.7–4.1). Thus, potential task effects on attentional orienting in the current study could not be due to confounds in emotional quality.

After the experiment, the participants filled in a number of questionnaires. These questionnaire data will be merged across different studies and are not reported within this manuscript.

### 2.5. Data processing and analysis

We used R (www.r-project.org) and Matlab^®^ R2014a (Mathworks, Inc., Natick, MA, USA) for data processing and analysis. In the majority of steps, the approach of one of our previous studies was followed closely (for further details, see [10]).

First, the graph-based visual saliency (GBVS) model [55] was used to create a saliency map for each scene (figure 1*b*,*c*) because it was previously shown to be among the most successful saliency-based gaze predictors which are biologically plausible and operate without initial machine learning ([36,38,55]; see also the MIT saliency benchmark [56]). However, all major results of the current study were qualitatively similar when using either the traditional Itti and Koch model [34,35] or a recent model (Boolean Map Saliency [57]) which performed very well on the MIT saliency benchmark [56]. The GBVS algorithm uses graph theory to reveal those locations of a visual stimulus that are most different from their surrounding in terms of three low-level features (i.e. colour, intensity and orientation). First conspicuous locations are calculated with respect to various spatial scales (i.e. 0.5, 0.25 and 0.125 of the original image's resolution) separately for each feature. Afterwards, all distributions are combined into an overall saliency map by first averaging the distributions for each feature and subsequently across all features. For this purpose, all distributions have to be represented in a common reference system. Therefore, the algorithm temporarily operates on the pixel level of a downscaled resolution (i.e. 0.027 of the original image's resolution) which is lower than the lowest spatial scale mentioned above. Finally, the overall saliency map is resized to the original image resolution.

Second, we determined four regions of interest (ROIs) for each scene (figure 1*b*,*c*). In a first step, the two social features heads and bodies were delineated manually using GIMP. On average, heads covered 2.1% of an image (s.d. = 2.5%, range = 0.1–10.2%) and bodies covered 8.9% (s.d. = 8.2%, range = 0.3–33.0%). In a second step, the saliency map was taken into account for those scene regions which had not already been classified as social ROIs and all parts with saliency values smaller or equal to the eighth saliency decile were defined as areas of lower saliency and the remaining regions as areas of higher saliency. On average, areas of lower saliency covered 71.3% of an image (s.d. = 8.3%, range = 49.6–79.9%) and areas of higher saliency covered 17.6% (s.d. = 2.1%, range = 12.2–19.9%). Although using the eighth saliency decile as cut-off was arbitrary, it served the purpose of identifying non-social scene parts with very high physical saliency, which was a necessary prerequisite for disentangling potential effects of social attention from physical saliency. The usefulness of this cut-off was further confirmed by using the ROIs of this stimulus set together with the saliency maps to further analyse the relative amount of physical saliency per ROI (see [10]). In detail, for each scene, the relative amount of physical saliency of each ROI was calculated by dividing the mean saliency per ROI by the mean saliency of the whole scene. This transformation permits a better comparison between different images and it allows for determining whether the saliency of a given ROI was above (i.e. values above 1) or below (i.e. values below 1) the average physical saliency of the image. Afterwards, these data were subjected to a one-way analysis of variance (ANOVA) with repeated measurements on the factor ROI (head, body, area of lower saliency, area of higher saliency). This analysis revealed a significant main effect of ROI, *F*_3,237_ = 153.91, *ɛ* = 0.63, *p* < 0.001, $\eta_{p}^{2}=0.66$, demonstrating that the areas of higher saliency (*M* = 2.19, s.e.m. = 0.03, range = 1.58–2.95) were more salient than the social ROIs (head: *M* = 1.59, s.e.m. = 0.08, range = 0.26–3.79; body: *M* = 1.51, s.e.m. = 0.08, range = 0.35–3.68) which were, in turn, more salient than the areas of lower saliency (*M* = 0.65, s.e.m. = 0.01, range = 0.41–0.93). Paired *post hoc t*-tests (two-tailed) indicated that the contrast between the social ROIs was not significant, *t*_79_ = 1.21, *p* = 0.23, while all other possible pairwise comparisons, all *t*_79_ > 7, all *p* < 10^−10^, reached statistical significance on a Bonferroni-corrected level (*α* = 0.05/6 = 0.0083).^3^ In addition, it was ensured that the social ROIs were not systematically located in shorter distance to scene centre than the areas of higher saliency (see [10]) to avoid potential effects of social attention as compared to physical saliency to be confounded with the typically occurring central fixation bias (e.g. [41,58–61]).

Third, we used the EyeLink's (SR Research, Ottawa, ON, Canada) standard parser configuration to detect saccades from participants' eye movements with a velocity threshold of 30° s^−1^ as well as an acceleration threshold of 8000° s^−2^. The time periods between saccades were classified as fixations. For each participant and trial, fixations which started after scene onset and before scene offset were considered for further data processing and entered drift correction with respect to a 300 ms baseline interval directly before scene onset (i.e. when the central cross was fixated). To prevent erroneous drift corrections for trials where participants failed to fixate the central fixation cross before stimulus onset, we accomplished a recursive outlier detection and removal procedure to each participant's distribution of baseline position data across all trials (see also [10,16,27]). In detail, separately for *x* and *y* coordinates, the minimum and maximum values were temporarily taken out of the respective distribution. If any of those two extreme values was located more than three standard deviations below or above the mean of the remaining distribution, it was discarded permanently. This procedure was iteratively applied to the minimum and maximum values of the resulting distribution until no more extreme values met the removal criterion. Finally, all trials with discarded or missing baseline position data were excluded from further data processing because participants most probably deviated from central fixation before scene onset in those trials. In addition, we considered only those trials in which less than 20% of the scene presentation duration were contaminated by blinks (defined by the EyeLink system as periods of missing pupil data). After discarding trials according to all mentioned exclusion criteria, a median of 39 trials per participant (s.d. = 2.4, range = 31–40) for free viewing and a median of 39 trials per participant (s.d. = 2.8, range = 29–40) for social detection were available for analysis.

Fourth, for each participant and trial, the drift-corrected fixations were used in combination with the ROIs to determine for each of the first five fixations on a scene which ROI was looked at. The analysis of the first five fixations particularly targeted the influences on very early eye movements because participants made on average 32.1 (s.d. = 3.5) fixations on a scene. Subsequently, for each participant and for each of the first five fixations, separately for both task blocks, the relative frequency that each ROI was fixated across all trials was calculated by dividing the frequency that each ROI was fixated by the frequency that any ROI was fixated. Because each scene's entire surface was assigned to the corresponding ROIs, the respective frequency that any ROI was fixated was identical to the number of trials with a valid scene fixation at the respective fixation number and task block. For normalization purposes, each relative frequency score was divided by the average area of the respective ROI across all trials which contributed data to the respective relative frequency score. Thus, it was controlled for the fact that the probability of receiving a fixation is higher for larger than smaller areas (e.g. [5,10,22,27]). Finally, these data were subjected to a three-way analysis of variance (ANOVA) with repeated measurements on the factors task (free viewing, social detection), ROI (head, body, area of lower saliency, area of higher saliency) and fixation number (1, 2, 3, 4, 5).

Last, for each participant and trial, we considered the drift-corrected fixations as well as the ROIs to specify the latency with regard to scene onset (in milliseconds) that each ROI was fixated for the first time. Next, for each participant and ROI, the median of these latency values was calculated across all trials with at least one valid fixation on the respective ROI, separately for both task blocks. Subsequently, a two-way ANOVA with repeated measurements on the factors task (free viewing, social detection) and ROI (head, body, area of lower saliency, area of higher saliency) was performed on these data. The numbers of trials per participant with at least one valid fixation on the respective ROI were as follows: head (free viewing: median = 33, s.d. = 4.0, range = 22–39; social detection: median = 34, s.d. = 4.0, range = 21–40), body (free viewing: median = 34, s.d. = 3.7, range = 25–40; social detection: median = 35, s.d. = 2.8, range = 28–40), area of lower saliency (free viewing: median = 38, s.d. = 2.7, range = 30–40; social detection: median = 39, s.d. = 2.9, range = 29–40), and area of higher saliency (free viewing: median = 38, s.d. = 2.6, range = 29–40; social detection: median = 38, s.d. = 2.9, range = 29–40).

The *a priori* significance level was defined as *α* = 0.05 throughout. However, Cramer *et al*. [62] recently emphasized that it is not only necessary to control for the inflated probability of Type-I errors when carrying out multiple *post hoc* comparisons subsequently to an ANOVA, but also when evaluating the main effects and interactions of an exploratory multiway ANOVA itself. Therefore, a Bonferroni-correction was applied to the *α* level of each multiway ANOVA and each set of multiple *post hoc* comparisons. For each analysis, the respective Bonferroni-corrected *α* level can be found in the Results section. For all repeated-measures ANOVAs including factors with more than one numerator degree of freedom, we report Huynh–Feldt's *ɛ* and corrected *p*-values to account for potential violations of the sphericity assumption. The ANOVAs were calculated with the help of the R package car (v. 2.0-21 [63]). All aggregated data and analysis scripts that are necessary for reproducing the present study's results are available as electronic supplementary material (see statement on data accessibility below).

## 3. Results

### 3.1. Fixation time course

**Figure 2.**
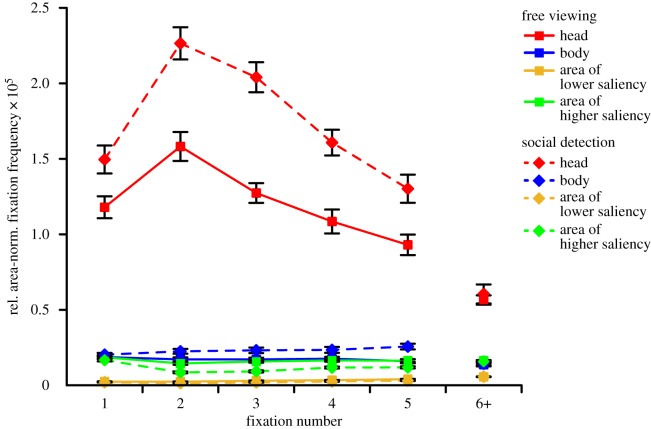
Fixation time course. Relative area-normalized frequency of each region of interest (ROI) being fixated at each of the first five fixated scene locations, separately for the two task conditions (free viewing versus spotting people as fast as possible). Note: data points represent the corresponding mean value, error bars denote s.e.m. ‘6+’ indicates the average value of the sixth to the last fixation.

**Figure 3.**
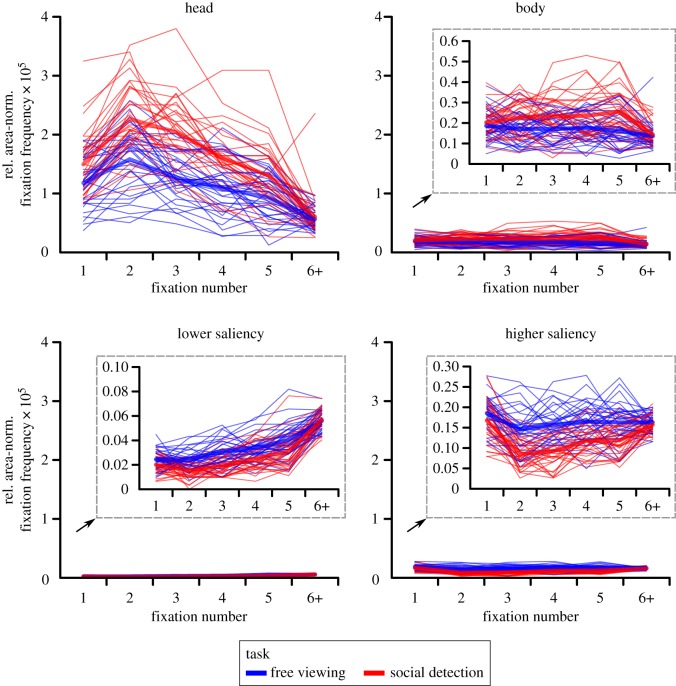
Fixation time course—individual participant scores. Relative area-normalized frequency of each region of interest (ROI) being fixated at each of the first five fixated scene locations, separately for the two task conditions (free viewing versus spotting people as fast as possible). Each line represents the individual scores of one participant in the corresponding condition. Thick lines denote the corresponding mean values. ‘6+’ indicates the average value of the sixth to the last fixation. For reasons of visibility, the scores for three ROIs (i.e. body, area of lower saliency and area of higher saliency) are additionally illustrated with scales zoomed in to the corresponding ranges.

The three-way ANOVA (Bonferroni-corrected *α* = 0.05/7 = 0.0071)^4^ on the relative area-normalized fixation frequency with repeated measurements on the factors task (free viewing, social detection), ROI (head, body, area of lower saliency, area of higher saliency) and fixation number (1, 2, 3, 4, 5) revealed significant main effects of task, *F*_1,32_ = 58.31, *p* < 0.001, $\eta_{p}^{2}=0.65$ (free viewing: *M* = 0.39 × 10^−5^, s.e.m. = 0.01×10^−5^; social detection: *M* = 0.53 × 10^−5^, s.e.m. = 0.02 × 10^−5^), and fixation number, *F*_4,128_ = 45.03, *ɛ* = 0.61, *p* < 0.001, $\eta_{p}^{2}=0.58$ (fixation 1: *M* = 0.43 × 10^−5^, s.e.m. = 0.02 × 10^−5^; fixation 2: *M* = 0.56 × 10^−5^, s.e.m. = 0.02 × 10^−5^; fixation 3: *M* = 0.50 × 10^−5^, s.e.m. = 0.02 × 10^−5^; fixation 4: *M* = 0.43 ×10^−5^, s.e.m. = 0.01 × 10^−5^; fixation 5: *M* = 0.38 × 10^−5^, s.e.m. = 0.01 × 10^−5^), as well as a significant interaction of task and fixation number, *F*_4,128_ = 6.51, *ɛ* = 0.88, *p* < 0.001, $\eta_{p}^{2}=0.17$. This indicated the average of the relative area-normalized fixation frequency across ROIs to vary between the first five fixated locations, depending on task. In addition, we found a significant main effect of ROI, *F*_3,96_ = 542.67, *ɛ* = 0.35, *p* < 0.001, $\eta_{p}^{2}=0.94$, representing heads to be fixated the most, followed by bodies which were looked at more than areas of higher saliency which were, in turn, fixated more than areas of lower saliency (figure 2). Paired *post hoc t*-tests (two-tailed; Bonferroni-corrected *α* = 0.05/6 = 0.0083) revealed all pairwise comparisons of ROIs to be significant, all *t*_32_ > 6.80, all *p* < 10^−7^. A visual inspection of the distributions of individual participant scores for the four ROIs, separately for each task and fixation number (figure 3), showed the individual scores for heads to be distinctly larger than the ones for all other ROIs with almost no overlap of ranges of scores. This indicated a large effect. By contrast, the ranges of scores for bodies and areas of higher saliency showed a large overlap, even though a substantial number of individual scores for bodies was above the range of scores for areas of higher saliency. This was particularly the case for the social detection condition where between 5 and 25 of 33 data points across all fixations on bodies exceeded the range of values for high saliency fixations. The individual scores for areas of lower saliency were clearly smaller than the ones for all other ROIs with only minimal overlap of ranges of scores.

Moreover, we observed a significant interaction of ROI and fixation number, *F*_12,384_ = 37.62, *ɛ* = 0.22, *p* < 0.001, $\eta_{p}^{2}=0.54$, indicating that the preferential fixation of heads was strongest for the earliest fixations, had its peak at the second fixation, and subsequently decreased from fixation to fixation. Additionally, we found a significant interaction of task and ROI, *F*_3,96_ = 58.15, *ɛ* = 0.37, *p* < 0.001, $\eta_{p}^{2}=0.65$, representing task to have different effects per ROI. Whereas the social features heads and bodies were fixated more in the social detection task as compared to free viewing (task difference for heads: *M* = 5.32 × 10^−6^, s.e.m. = 0.69 × 10^−6^; task difference for bodies: *M* = 5.70 × 10^−7^, s.e.m. = 1.50 × 10^−7^), the pattern was reversed both for areas of lower and higher saliency (task difference for areas of lower saliency: *M* = −8.43 × 10^−8^, s.e.m. = 1.17 × 10^−8^; task difference for areas of higher saliency: *M* = −4.68 × 10^−7^, s.e.m. = 0.62 × 10^−7^). Paired *post hoc t*-tests (two-tailed; Bonferroni-corrected *α* = 0.05/4 = 0.0125) revealed the comparison of both task blocks to be significant for each ROI, all *t*_32_ > 3.75, all *p* < 0.001. Visually inspecting the distributions of individual participant scores for the four ROIs, separately for each task and fixation number (figure 3), revealed that the ranges of scores for all ROIs partially overlapped between both task blocks. Beyond this overlap, for heads, a substantial number of individual scores for social detection was above the overlapping range (i.e. between 4 and 18 of 33 data points across all fixations). For the other ROIs, the overlap was relatively larger and the number of individual scores falling outside of the common range relatively smaller. This demonstrated that the task effect was largest for heads.

Finally, we observed a significant three-way interaction of task, ROI and fixation number, *F*_12,384_ = 5.56, *ɛ* = 0.32, *p* < 0.001, $\eta_{p}^{2}=0.15$. This interaction indicated that the effects of task on each ROI varied between the first five fixated locations with effects being most pronounced for the second and third fixation, especially for heads. Following this interaction, because of the central importance of the very first fixation for the current study's research question, we calculated a *post hoc* two-way ANOVA (Bonferroni-corrected *α* = 0.05/3 = 0.0167)^5^ with repeated measurements on the factors task (free viewing, social detection) and ROI (head, body, area of lower saliency, area of higher saliency) for the very first fixation. This analysis revealed significant main effects of task, *F*_1,32_ = 12.66, *p* = 0.001, $\eta_{p}^{2}=0.28$, and ROI, *F*_3,96_ = 268.89, *ɛ* = 0.35, *p* < 0.001, $\eta_{p}^{2}=0.89$, as well as a significant interaction of task and ROI, *F*_3,96_ = 12.08, *ɛ* = 0.38, *p* < 0.001, $\eta_{p}^{2}=0.27$. Subsequently, paired *post hoc t*-tests (two-tailed; Bonferroni-corrected *α* = 0.05/4 = 0.0125) for the very first fixation revealed a significant difference between both task blocks only for heads, *t*_32_ = 3.58, *p* = 0.001 (task difference: *M* = 3.16 × 10^−6^, s.e.m. = 0.88 × 10^−6^),^6^ while this contrast did not reach statistical significance for the other ROIs on the adjusted level, all *t*_32_ < 2.30, all *p* > 0.025. Figure 5*a* further illustrates the individual participant scores for heads at the very first fixation as paired observations between both task blocks. A visual inspection of this figure indicated a large and relatively consistent task effect: whereas the majority of 25 participants exhibited larger individual scores in the social detection task when compared to free viewing in accordance with the overall sample statistics (one of them very subtle), a minority of eight volunteers showed an effect in the opposite direction. Furthermore, there were participants exhibiting larger scores for social detection as compared to free viewing almost across the whole range of individual scores. Participants were an exception who already had the largest individual scores for the free viewing condition.

### 3.2. Latency analysis

**Figure 4.**
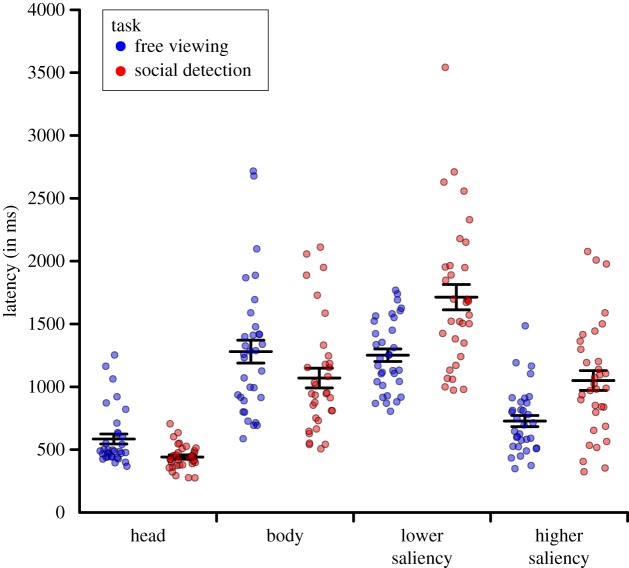
Latency. Median latency after stimulus onset (in milliseconds) until each region of interest (ROI) was fixated for the first time, separately for the two task conditions (free viewing versus spotting people as fast as possible). Each point represents one participant. Points were jittered to reduce overlap. The long black horizontal bars indicate the corresponding mean values, the surrounding black error bars denote s.e.m.

In the two-way ANOVA (Bonferroni-corrected *α* = 0.05/3 = 0.0167) on the median latency of image regions being fixated for the first time with repeated measurements on the factors task (free viewing, social detection) and ROI (head, body, area of lower saliency, area of higher saliency), we observed a significant main effect of ROI, *F*_3,96_ = 61.21, *ɛ* = 0.89, *p* < 0.001, $\eta_{p}^{2}=0.66$. This effect indicated that, in the case that the respective ROI was fixated, heads were looked at fastest, followed by areas of higher saliency which were looked at faster than bodies which were, in turn, looked at faster than areas of lower saliency (figure 4). Paired *post hoc t*-tests (two-tailed; Bonferroni-corrected *α* = 0.05/6 = 0.0083) revealed that all pairwise comparisons of ROIs were significant, all *t*_32_ > 3.30, all *p* < 0.003. Moreover, visually inspecting figure 4 with respect to the distributions of individual participant scores for the four ROIs, separately for each task, showed the range of individual scores for heads to be distinctly smaller than the ranges for all other ROIs. Additionally, the majority of individual scores for heads were smaller than a substantial number of individual scores for areas of higher saliency, even though the scores for heads largely lay within the range of possible scores for areas of higher saliency. Furthermore, the majority of individual scores for heads were smaller than the majority of individual scores for bodies (only little overlap between distributions) as well as areas of lower saliency (almost no overlap between distributions) indicating a large effect.

**Figure 5.**
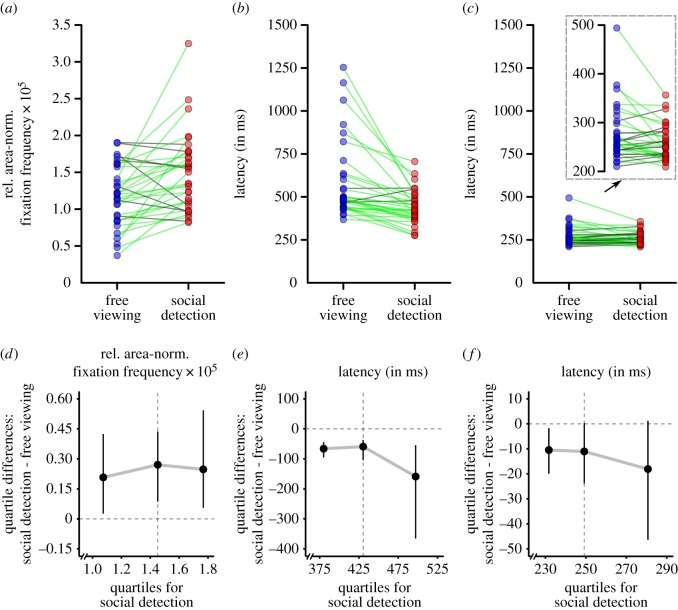
Paired observations and shift functions. Top row: individual participant scores illustrated as paired observations between the two task conditions (free viewing versus spotting people as fast as possible) for three measures: (*a*) the relative area-normalized frequency of heads being fixated at the very first fixation after scene onset, (*b*) the median latency (in milliseconds) of heads being fixated for the first time, and (*c*) the median latency (in milliseconds) of fixating heads at the very first fixation after scene onset. Note: green links indicate paired observations with a numerical task difference in accordance with the overall sample statistics (see Results section); grey lines denote a reversed numerical task difference. For reasons of visibility, the scores for (*c*) are additionally illustrated with scales zoomed in to the corresponding ranges. Bottom row: shift functions (see Results section) for the difference in the distributions of individual participant scores between the two task conditions (free viewing versus spotting people as fast as possible) for three measures: (*d*) the relative area-normalized frequency of heads being fixated at the very first fixation after scene onset, (*e*) the median latency (in milliseconds) of heads being fixated for the first time, and (*f*) the median latency (in milliseconds) of fixating heads at the very first fixation after scene onset. Note: the shift functions display the differences between the quartiles for social detection and free viewing (*y*-axes) as a function of the first, second and third quartile for social detection (*x*-axes). Consequently, for each quartile of social detection, it is illustrated in which way and to which degree the corresponding quartile for free viewing would need to be shifted to match the corresponding quartile for social detection (see also [64]). Vertical bars denote 95% bootstrap confidence intervals. Vertical dashed lines mark the second quartiles (i.e. medians).

In addition, even though the main effect of task did not reach statistical significance on the adjusted level, *F*_1,32_ = 5.40, *p* = 0.027, $\eta_{p}^{2}=0.14$, we found a significant interaction of task and ROI, *F*_3,96_ = 16.58, *ɛ* = 0.68, *p* < 0.001, $\eta_{p}^{2}=0.34$, representing task to have different effects per ROI. In the case that the respective ROI was fixated, the social features heads and bodies were looked at faster in the social detection task as compared to free viewing (task difference for heads: *M* = −143.32 ms, s.e.m. = 31.89 ms; task difference for bodies: *M* = −210.12 ms, s.e.m. = 113.28 ms), while the pattern was reversed both for areas of lower and higher saliency (task difference for areas of lower saliency: *M* = 462.03 ms, s.e.m. = 97.78 ms; task difference for areas of higher saliency: *M* = 322.65 ms, s.e.m. = 73.79 ms). Paired *post hoc t*-tests (two-tailed; Bonferroni-corrected *α* = 0.05/4 = 0.0125) revealed the comparison of both task blocks to be significant for heads as well as areas of lower and higher saliency, all *t*_32_ > 4.35, all *p* < 0.001, while this contrast did not reach statistical significance for bodies, *t*_32_ = 1.86, *p* = 0.073. Figure 5*b* further illustrates the individual participant scores for heads as paired observations between both task blocks. Visually inspecting this figure demonstrated a large and relatively consistent task effect: 30 participants had smaller individual scores in the social detection task as compared to free viewing in line with the overall sample statistics (four of them very subtle), while only three volunteers showed a reversed pattern (two of them very subtle). There were participants exhibiting smaller scores for social detection as compared to free viewing across the whole range of individual scores. Moreover, this task effect was larger for participants with large scores for free viewing than for participants with small or medium scores for free viewing.

Following up on the effect that the median latency of heads being fixated for the first time was shorter in the social detection task as compared to free viewing and because of this result's central importance for the current study's research question, we carried out an additional *post hoc* analysis. The above-reported analyses were based on the latency until heads were fixated for the first time in a trial regardless of the fixation number after scene onset this fixation occurred. Therefore, in an additional paired *post hoc t*-test (two-tailed), we again compared the median latency of heads being fixated for the first time between both task blocks, but this time considered only those trials in which heads, in fact, received the first fixation after scene onset. This analysis revealed a significant difference between both task blocks, *t*_32_ = 2.69, *p* = 0.011 (task difference: *M* = −18.03 ms, s.e.m. = 6.70 ms), indicating that if heads were fixated at the very first fixation after scene onset, the latency of this fixation was shorter in the social detection task (*M* = 258.59 ms, s.e.m. = 6.31 ms) as compared to free viewing (*M* = 276.62 ms, s.e.m. = 9.91 ms). Figure 5*c* further illustrates the corresponding individual participant scores as paired observations between both task blocks. A visual inspection of this figure showed that the majority of 22 participants exhibited smaller individual scores in the social detection task as compared to free viewing in accordance with the overall sample statistics (four of them very subtle), while a minority of 11 volunteers showed the opposite pattern (two of them very subtle). The task effect of smaller individual scores for social detection (versus free viewing) was larger for participants with large scores for free viewing than for participants with small or medium scores for free viewing. Furthermore, only participants with small and medium scores for free viewing exhibited the reversed task effect (i.e. larger individual scores for social detection versus free viewing). Finally, visually inspecting figure 5*c* demonstrated that the individual scores for social detection largely lay in the range of possible scores for free viewing.

### 3.3. Shift functions

The analyses described above revealed task effects on three indicators of rapid orienting towards heads: (i) the relative area-normalized fixation frequency on heads at the first fixation after scene onset, (ii) the median latency of heads being fixated for the first time and (iii) the median latency of heads being fixated for the first time considering only trials in which heads received the first fixation after scene onset. However, even though we displayed and inspected individual participant scores and paired observations in figures, our statistical analyses were so far based on the standard approach in psychological and neuroscientific research: We investigated the existence of task effects solely by comparing the central tendency (i.e. the means) of the distributions of participant scores. Importantly, Rousselet *et al*. [64] recently emphasized the potential that lies within comparing two distributions more comprehensively by means of so-called shift functions (see also e.g. [65,66]). A shift function is both a graphical and an inferential method to analyse in which way the quantiles (e.g. the first, second and third quartile) of one distribution would need to be shifted to match the corresponding quantiles of another distribution. Crucially, this technique facilitates the comparison of two distributions beyond measures of central tendency (e.g. the medians/second quartiles) by also analysing how the distributions differ in areas located further away from the distribution centres (e.g. the first and third quartiles) [64]. For this purpose, we built a *post hoc* shift function for each of the three described indicators of rapid orienting towards heads to quantify more comprehensively how the distributions of participant scores differed between free viewing and social detection. This was accomplished by means of the R package rogme (v. 0.2.0 [67]). In detail, for each of the three indicators, the Harrell–Davis quantile estimator [68] was used to estimate the first, second and third quartiles of the two distributions. To facilitate the use of these specific quantiles, we implemented a recently proposed modified version of the shift function for dependent distributions [69]. For inferences, this version calculates 95% confidence intervals of the quantile differences using a straightforward percentile bootstrap of the quantiles (standard number of bootstrap samples = 1000) [64,67,69].

First, we built a *post hoc* shift function to compare the distributions of participant scores of the relative area-normalized fixation frequency on heads at the first fixation after scene onset between free viewing and social detection. Figure 5*d* shows this shift function by displaying the quartiles for social detection on the *x*-axis, the difference between the quartiles of social detection and free viewing on the *y*-axis, and the 95% confidence intervals of the quartile differences as error bars. All three quartile differences were found to be positive. This indicated that each quartile of social detection had a larger value than the corresponding quartile of free viewing (i.e. each quartile of free viewing would need to be shifted up to larger values to match the corresponding quartile of social detection). However, when evaluating the *p*-values of the three quartile differences on a Bonferroni-corrected *α* (0.05/3 = 0.0167), only the differences for the second (*p* = 0.004) and third quartile (*p* = 0.008) were significant, while the difference for the first quartile (*p* = 0.026) did not reach statistical significance on the adjusted level.^7^ Consequently, the distributions of participant scores of the relative area-normalized fixation frequency on heads at the first fixation after scene onset did not only differ between free viewing and social detection in central tendency, but also in areas located further away from the distribution centres (i.e. especially the third quartiles).

Second, we built a *post hoc* shift function to compare the distributions of participant scores of the median latency of heads being fixated for the first time between free viewing and social detection (figure 5*e*). All three quartile differences (social detection minus free viewing) were found to be negative. This demonstrated that each quartile of social detection had a smaller value than the corresponding quartile of free viewing (i.e. each quartile of free viewing would need to be shifted down to smaller values to match the corresponding quartile of social detection). All three quartile differences (first quartile: *p* < 0.001, second quartile: *p* < 0.001, third quartile: *p* = 0.002) were significant on a Bonferroni-corrected *α* (0.05/3 = 0.0167). The quartile difference was most negative for the third quartile, even though the 95% confidence interval indicated increased uncertainty. Altogether, this suggests that the distribution of participant scores of the median latency of heads being fixated for the first time was generally shifted down to smaller values for social detection when compared to free viewing. However, this downward shift was most pronounced for the areas above the distribution centres (i.e. the third quartiles).

Third, we built an identical *post hoc* shift function to compare the distributions of participant scores of the median latency of heads being fixated for the first time between free viewing and social detection, but this time considered only trials in which heads, in fact, received the first fixation after scene onset (figure 5*f*). In accordance with the previously reported shift function, all three quartile differences (social detection minus free viewing) were found to be negative. This reflected that each quartile of social detection had a smaller value than the corresponding quartile of free viewing (i.e. each quartile of free viewing would need to be shifted down to smaller values to match the corresponding quartile of social detection). Furthermore, the quartile difference was again more negative for the third quartile than for the first and the second ones. However, on a Bonferroni-corrected *α* (0.05/3 = 0.0167), none of the three quartile differences (first quartile: *p* = 0.022, second quartile: *p* = 0.062, third quartile: *p* = 0.080) reached statistical significance.

## 4. Discussion

The present study aimed at investigating whether the early bias of attentional orienting towards social features such as heads or bodies in visual scenes can be further accelerated by top-down instructions. Therefore, participants viewed complex naturalistic social scenes either freely or with the task to spot people in each image as fast as possible while their eye movements were recorded. In analyses of the first five fixated scene locations, we found a strong attentional preference for social features (especially heads) over physically salient image parts which was particularly pronounced for very early fixations. Moreover, this bias was increased when participants were instructed to detect people quickly as compared to free exploration. Importantly, this task-dependent enhancement of attending to social information (especially heads) was strongest for very early fixations (i.e. the second and third fixation) and already evident at the very first fixation after scene onset. Accordingly, latency analyses revealed that, if social features were looked at in a trial, the time until they (especially heads) were fixated for the first time was shorter in the social detection condition as compared to free viewing. Finally, even when we considered only those trials in which already the very first fixation after scene onset was directed towards heads, the latency of this first fixation was shorter when the participants' task was to spot people as fast as possible as compared to free exploration.

The current finding that the strong prioritization of social elements (especially heads) in naturalistic scenes was most pronounced for the very first fixations replicates previous studies (e.g. [10,12–16]) which indicated that a bias of orienting towards humans is present and potentially even strongest at very early viewing stages. This again demonstrates that the preference for social features in visual scenes does not just emerge after several seconds of visual exploration. Thus, the present study corroborates the notion that this social prioritization phenomenon at least partly relies on reflexive and automatic processes.

In addition, the current finding that the heavy prioritization of humans in complex scenes was more pronounced under social detection than free viewing conditions is in line with previous research (e.g. [18–24]) showing that the degree of orienting to social information can be modulated by task instructions. However, contrary to most prior studies in this domain, we conducted detailed analyses of the first five fixated scene locations as well as the latency until different scene regions were fixated for the first time. This enabled us to not only investigate top-down influences on social attention in general, but to especially shed light on the following question: Was the task-dependent enhancement of orienting to social elements (especially heads) in naturalistic scenes already present at the very first fixation? Importantly, previous research on this issue is sparse and partly yielded inconsistent results. On the one hand, Fletcher-Watson and colleagues [29,30] presented their participants with composite stimuli comprising a social and a non-social scene. This research yielded evidence that observers directed more first fixations to the social stimulus side and particularly to a depicted person when their task was to discriminate gender than when they freely viewed the visual material. On the other hand, Birmingham *et al*. [28] did not find support for an increased early orienting to human eyes in visual scenes when the task was to determine where depicted people directed their attention to as compared to general image description or free viewing tasks. Furthermore, another study found participants to direct more first fixations to human eyes in visual scenes when being informed about a subsequent image memory test. However, this task-dependent increase in early social attention did not concern the social feature most preferred at first fixations. In detail, heads exceeded eyes at this early viewing stage but received even less first fixations when observers were informed about the following image recognition test (early fixation data of [71] reported in [12]).

Moreover, the few other studies touching the issue of top-down-generated increase of rapid orienting to social elements in visual scenes (e.g. [72,73]) mostly either did not find conclusive task effects on social attention at early viewing stages or did not sufficiently compare tasks intended to improve social attention with genuine free viewing conditions. Therefore, the current study makes a substantial contribution to social attention research by providing clear evidence that the instruction to spot people as fast as possible can additionally enhance the rapid preference for social features (especially heads) in naturalistic scenes as compared to free exploration at the very first fixation. Importantly, we demonstrate explicitly that this top-down manipulation can not only enhance the amount of first fixations on heads, but also accelerate the speed with which first fixations are directed to heads. Thus, it can be excluded that the task-dependent enhancement of early social attention emerged due to a speed–accuracy trade-off. For instance, this trade-off would have been indicated by more first fixations directed to social features at the expense of taking more time to execute the corresponding saccades.

Furthermore, a detailed analysis of individual participant scores (e.g. stripcharts of paired observations) and so-called shift functions (e.g. [64]) revealed that the task-dependent acceleration of attending to heads was most pronounced for individuals who oriented towards heads only relatively slowly under free viewing conditions as compared to the rest of the sample. By contrast, individuals who already were among the fastest in allocating attention to heads during free viewing could not speed up this social orienting process to the same degree. In addition, there seemed to be something like a lower boundary at around 210 ms for the median latency of fixations targeting heads immediately after scene onset. Accordingly, none of the participants exhibited a substantially faster median latency of directing first fixations to heads in the social detection task than 210 ms. Crucially, this was also the lower boundary for the median latency of directing first fixations to heads under free viewing conditions. Thus, whereas the majority of participants were able to additionally accelerate the rapid orienting towards heads by means of top-down instructions, it might have been particularly difficult for the fastest participants to further speed up social attention.

By systematically considering low-level stimulus properties, the present study facilitated disentangling the influence of social attention from potential effects of physical saliency. In contrast to the majority of previous studies, we took care during stimulus selection that social elements were not confounded by physical saliency. Furthermore, we explicitly verified that social features were visual elements with only intermediate low-level saliency. On this basis, we compared to which degree social features and non-social areas with highest physical saliency received early fixations. Importantly, this ensured that the observed impact of task instructions on rapidly attending towards social features was indeed mediated by top-down influences which acted upon the processing of social information and not simply by modulations of the sensitivity to physically salient image parts. Hence, the current study particularly extends prior research by two combined findings: first by demonstrating that the instruction to spot depicted people as fast as possible can enhance the rapid preference for social features (especially heads) as compared to free viewing at the very first fixation; second, by showing that this is valid even if physically more conspicuous non-social information is present in the visual field. Therefore, our findings strongly indicate that it will be essential for future theories of human visual attention to integrate not only top-down demands and bottom-up processing of stimulus characteristics, but also social influences as well as interactions between these factors (see also [10,27,74]).

Furthermore, it is a crucial feature of the present study that we implemented the direct task to spot people in naturalistic scenes as fast as possible. A few previous studies have already used tasks that directly included to search for a predefined face exemplar or to make speeded saccades to faces in visual scenes (e.g. [73,75]). However, these reports did mostly not include systematic comparisons of the degree that first fixations were directed to social elements between face search tasks and conditions of free exploration. Thus, despite providing evidence that attention can be directed to certain high-level stimuli such as faces in a very fast manner, these studies cannot answer the question of whether the rapid orienting towards social information was faster than when the visual material would have been looked at freely. Contrary to this, several prior studies investigating top-down influences on attending to social elements in visual scenes made comparisons to free exploration conditions (e.g. [28–30]). However, these studies typically did not contain direct task instructions to fixate depicted people quickly but rather indirectly required to attend to them (e.g. by using a gender discrimination task or by asking to determine the attentional focus of depicted individuals). Whereas these instructions were most suitable for the specific research questions of those studies, they did not aim at maximizing the chance of causing task-dependent enhancements of social attention at very early fixations. Therefore, one cannot conclude from the absence of top-down modulations of early eye movements to social information in some previous studies that it is impossible to further speed up social attention. Importantly, even though a similar research approach has already been suggested by other authors (e.g. [30]), to the best of our knowledge, the present study is the first to implement the direct instruction to spot people in ecologically valid naturalistic scenes as fast as possible for the purpose of investigating the research question: Can the preferential orienting of first fixations to social elements be additionally enhanced as compared to free viewing? Future research should try to apply this approach to populations and circumstances for which task-dependent modulations of social attention have previously been reported to be relatively weak or absent, for example to individuals with autism spectrum disorders (cf. [72]).

In addition to the strengths of the current study, some shortcomings should also be discussed. First, we always applied the two task blocks in the same order, namely first viewing the stimuli freely and afterwards spotting people as fast as possible. On the one hand, the implementation of this fixed task order instead of balancing block order between observers or varying the task in a trial-by-trial manner had the advantage of avoiding potential carry-over effects from the specific top-down demands of the social detection task to free exploration. Specifically, this ruled out the risk that as soon as participants know about the particular priority of social features for one task, they might involuntarily transfer it to other conditions. On the other hand, the drawback of always applying the two task blocks in the same order was that, even though potentially relevant aspects were controlled (e.g. by randomizing the assignment of scenes to task blocks as well as the order of stimuli), one cannot exclude that other factors might have differed between the first and the second half of the experiment, for example fatigue or practice. We did not find any indication that participants were more tired in the second experimental phase (e.g. no overall increase in the latency until each scene region was fixated for the first time). Furthermore, increased and accelerated orienting towards heads in the social detection task, as compared to free viewing, was stable between the first and the second halves within each block (see electronic supplementary material on distinguishing task from practice effects, section S1 and table S1). This indicates that the currently observed scanning pattern does not seem to rely on unspecific practice or fatigue effects. Nevertheless, future research should try to test whether the current findings can be replicated when task order is balanced between subjects or varies from trial to trial. Second, we evaluated the potential of top-down instructions to enhance early social attention in complex scenes by using free exploration as a baseline because this condition is typically thought to basically resemble some kind of natural viewing mode (e.g. [40]). However, free viewing remains a specific task employed in laboratory settings and one can neither verify if people's gaze behaviour really corresponds to unconstrained real-world viewing nor control what individual strategies are actually applied by each observer (see [41,76]). Thus, future studies should try to explore whether the effects observed in the present study can be confirmed when the task to detect people quickly is contrasted with different baseline instructions. Third, the task-dependent acceleration of the median latency of fixating heads at the very first fixation after scene onset just failed to reach statistical significance on a very conservatively corrected level in some *post hoc* analyses (i.e. the analysis of shift functions and the analysis for distinguishing task from practice effects in the electronic supplementary material, section S1 and table S1). However, these analyses were rather supplementary in the context of the present study and the task-dependent acceleration of social attention was descriptively clearly evident in these analyses. In addition, this task effect was evident in the majority of our participants and reached statistical significance in the primary *post hoc* comparison investigating it. This statistically significant effect was a necessary prerequisite for the mentioned supplementary analyses in the first place. Thus, we are confident in interpreting that top-down instructions indeed have the potential to accelerate the speed with which first fixations are directed to heads as compared to free exploration. Nevertheless, future research should try to replicate this finding. Fourth, even though we presented observers with ecologically valid naturalistic scenes instead of impoverished stimuli such as isolated faces, our study was still conducted in a controlled laboratory setting which can never be an exact representation of real-world situations (see also [7]). Interestingly, several recent studies (e.g. [77–80]) yielded support that social attention in real-world circumstances may be modulated by top-down demands arising from different tasks (e.g. speaking or listening to another person) or by social context (e.g. eating or sitting in waiting room). However, the majority of prior studies did not specifically focus on very early viewing stages. Therefore, future research will have to investigate whether the task-dependent enhancements of rapid social attention observed in the present study can be confirmed when observers' eye movements are recorded in real-world social situations (see also [7]).

In conclusion, the current study makes a substantial contribution to the characterization of the nature of the mechanisms underlying rapid social attention. We replicate that humans heavily prioritize other people (especially heads) in the visual field irrespective of their low-level saliency and that this effect is most pronounced for the earliest fixations. Moreover, our findings also demonstrate that the rapid preference for social features (especially heads) in naturalistic visual material can be additionally enhanced when observers are directly instructed to spot people as quickly as possible. This corroborates the idea that attentional orienting towards social elements in our environment not only relies on volitionally controlled mechanisms, but especially on reflexive and automatic processes. However, most importantly, it provides strong evidence that these rapid processes can be additionally accelerated by top-down instructions.

## Supplementary Material

Supplementary sections and tables

## Footnotes

1 Please note that the terms ‘direct’ and ‘indirect’ at this point do not refer to the dissociation of overt and covert aspects of attention.

2 Please note that in the case of dependent (i.e. probably correlated) distributions, the effect size estimate is calculated differently than for independent distributions and is typically referred to as Cohen's *d_z_* (see [48]).

3 The results of this control analysis were previously reported in the study of End and Gamer (2017, see fig. 3*b*) [10]. In addition, please note that calculating the relative saliency scores for each scene with respect to the maximum saliency of each region of interest (ROI) instead of the mean saliency of each ROI revealed similar results with social ROIs being of intermediate physical saliency (i.e. significantly less salient than areas of higher saliency, but significantly more salient than areas of lower saliency).

4 The significance level of the three-way ANOVA was adjusted to α = 0.05/7 = 0.0071 because this analysis comprised seven hypotheses, i.e. three main effects, three two-way interactions and one three-way interaction (see [62]).

5 The significance level of the two-way ANOVA was adjusted to α = 0.05/3 = 0.0167 because this analysis comprised three hypotheses, i.e. two main effects and one interaction (see [62]).

6 A similar effect was also observed in raw relative fixation frequencies (i.e. without area-normalization) on heads at the very first fixation (reported in %): free viewing: *M* = 27.9%, s.e.m. = 1.6%, range = 7.5–43.6%; social detection: *M* = 33.8%, s.e.m. = 1.9%, range = 19.4–57.5%.

7 Please note that although significance levels for evaluating quartile differences in the context of shift functions are typically adjusted according to Hochberg's method ([70]; see [67]), we used the more conservative Bonferroni correction to be consistent with the other analyses in the present study.

## Data accessibility

The data and analysis scripts supporting the present study's results are available as electronic supplementary material. As the consent forms did not include a statement on the publication of individual participants' raw data, we only provide aggregated datasets. The stimulus material was selected from several stimulus sets (for a complete list, see electronic supplementary material, table S2) and the Internet. As we do not have permission for sharing these pictures with the article, they are not included in the electronic supplementary material.
