# Supplementary material for: Task instructions can accelerate the early preference for social features in naturalistic scenes
Source: R Soc Open Sci. 2019 Mar 6;6(3):180596. doi: 10.1098/rsos.180596 (PMC6458421; doi:10.1098/rsos.180596)
Supplement: Supplementary sections and tables [file rsos180596supp1.pdf]

Supplementary information:

Task instructions can accelerate the early preference for social features in naturalistic scenes

Albert End & Matthias Gamer

Section S1 | Distinguishing task from practice effects

As mentioned in the methods section, the two task blocks were applied in the same order for each participant (i.e., first free viewing, then social detection). This fixed task order was implemented in order to avoid potential carry-over effects from the specific top-down demands of the social detection task to free exploration (for a detailed comparison of the advantages and disadvantages of this study design, see the discussion section). However, in this context, it is necessary to rule out that the increased and accelerated attentional orienting to heads in the social detection task as compared to free viewing was driven by the fixed order of tasks instead of the actual tasks themselves. For example, an alternative interpretation of the described task effects might be that the participants' tendency to orient towards heads simply increased as a function of time because participants might get better in orienting to this type of information as they do more trials. To distinguish actual task from such practice effects, we performed additional post-hoc analyses of the three central indicators of task effects on rapid orienting towards heads by subdividing each task block into a corresponding first and second half. On the one hand, actual task effects should be characterized by a sudden increase and acceleration of the attentional orienting towards heads when the task changes between the two task blocks. On the other hand, practice effects would be rather indicated by a gradual increase and acceleration of the attentional orienting towards heads from each block half to the next one.

First, we performed a post-hoc two-way ANOVA (Bonferroni-corrected $\alpha = .05/3 = .0167$) on the relative area-normalized fixation frequency on heads at the very first fixation with repeated measurements on the factors task (free viewing, social detection) and block part (first half, second half). This analysis revealed a significant main effect of task, $F(1,32) = 10.68$, $p = .003$, $\eta^2_p = .25$, but neither a significant main effect of block part, $F(1,32) = 0.03$, $p = .87$, $\eta^2_p = .00$, nor a significant interaction of task and block part, $F(1,32) = 0.43$, $p = .52$, $\eta^2_p = .01$ (see Table S1). Second, we calculated a post-hoc two-way ANOVA (Bonferroni-corrected $\alpha = .05/3 = .0167$) on the median latency of heads being fixated for the first time with repeated measurements on the factors task (free viewing, social detection) and block part (first half, second half). This analysis revealed a significant main effect of task, $F(1,32) = 16.72$, $p < .001$, $\eta^2_p = .34$, but neither a significant main effect of block part, $F(1,32) = 0.00$, $p = .97$, $\eta^2_p = .00$, nor a significant interaction of task and block part, $F(1,32) = 1.12$, $p = .30$, $\eta^2_p = .03$ (see Table S1). Third, we calculated an identical post-hoc two-way ANOVA (Bonferroni-corrected $\alpha = .05/3 = .0167$) on the median latency of heads being fixated for the first time but this time considered only those trials in which heads in fact received the first fixation after scene onset. In this analysis, the main effect of task did not reach statistical significance on the adjusted level, $F(1,32) = 4.43$, $p = .043$, $\eta^2_p = .12$. Furthermore, there was neither a significant main effect of block part, $F(1,32) = 1.71$, $p = .20$, $\eta^2_p = .05$, nor a significant interaction of task and block part, $F(1,32) = 1.53$, $p = .22$, $\eta^2_p = .05$ (see Table S1).

Altogether, the three reported post-hoc analyses yielded strong evidence that the increased and accelerated attentional orienting towards heads in the social detection task as compared to free viewing was indeed driven by the actual tasks themselves. There was no evidence that these effects could be alternatively explained by practice effects occurring during the time course of the whole experiment.

Table S1 | Descriptive statistics for three indicators of rapid fixations on heads, separately for the first and second half of each task block (i.e., free viewing, social detection): Relative area-normalized fixation frequency ($\cdot 10^5$) on heads at the first fixation after scene onset, median latency (in ms) of heads being fixated for the first time, and median latency (in ms) of heads being fixated for the first time considering only trials in which heads received the first fixation after scene onset.

	Free viewing		Social detection	
	Block half 1	Block half 2	Block half 1	Block half 2
Rel. area-norm. fixation frequency $\cdot 10^5$ (fixation no. 1)				
M	1.18	1.25	1.55	1.51
SEM	0.10	0.10	0.12	0.12
Min	0.37	0.31	0.45	0.33
Max	2.62	2.52	3.94	3.01
Latency (in ms)				
M	627.7	663.5	465.5	432.9
SEM	52.3	74.8	25.3	23.5
Min	360.0	339.0	256.5	276.5
Max	1484.0	2541.5	880.0	847.0
Latency (in ms; fixation no. 1)				
M	274.8	275.0	265.7	255.6
SEM	8.8	10.1	6.9	6.5
Min	210.0	202.0	214.0	198.5
Max	442.5	494.0	371.0	380.0

Note. rel. = relative; area-norm. = area-normalized; no. = number; min = minimum; max = maximum.

Table S2 | List of 33 stimuli selected from four different stimulus databases. The remaining 47 stimuli were collected from the internet. Please note that the images were cropped or rescaled if necessary.

Stimulus name	Database
009	EmoPicS
119	EmoPicS
131	EmoPicS
133	EmoPicS
138	EmoPicS
191	EmoPicS
196	EmoPicS
197	EmoPicS
205	EmoPicS
5199	IAPS
9150	IAPS
merry_florida0011	McGill
merry_mexico0072	McGill
merry_mexico0143	McGill
pippin_city6	McGill
pippin_city66	McGill
Faces_023_h	NAPS
Faces_265_h	NAPS
Faces_290_h	NAPS
Faces_302_h	NAPS
People_009_h	NAPS
People_015_h	NAPS
People_022_h	NAPS
People_054_h	NAPS
People_058_h	NAPS
People_109_h	NAPS
People_116_h	NAPS
People_131_h	NAPS
People_157_h	NAPS
People_158_h	NAPS
People_167_h	NAPS
People_182_h	NAPS
People_195_h	NAPS

Note. EmoPicS = Emotional Picture Set [1];
IAPS = International Affective Picture System [2];
McGill = McGill Calibrated Colour Image Database [3];
NAPS = Nencki Affective Picture System [4].

Supplementary references

1. Wessa M, Kanske P, Neumeister P, Bode K, Heissler J, Schönfelder S. 2010 EmoPics: subjektive und psychophysiologische Evaluation emotionalen Bildmaterials zur klinischen und biopsychologischen Forschung. *Z. Für Klin. Psychol. Psychother. Suppl.* **1**, 77. (doi:10.1026/1616-3443/a000036)

2. Lang PJ, Bradley MM, Cuthbert BN. 2008 *International Affective Picture System (IAPS): Affective ratings of pictures and instruction manual*. Technical Report No. A-8. Gainesville, FL.: University of Florida.

3. Olmos A, Kingdom FAA. 2004 A biologically inspired algorithm for the recovery of shading and reflectance images. *Perception* **33**, 1463–1473. (doi:10.1068/p5321)

4. Marchewka A, Żurawski Ł, Jednoróg K, Grabowska A. 2014 The Nencki Affective Picture System (NAPS): introduction to a novel, standardized, wide-range, high-quality, realistic picture database. *Behav. Res. Methods* **46**, 596–610. (doi:10.3758/s13428-013-0379-1)
